# Cerebral pleomorphic xanthoastrocytoma mimicking inflammatory granuloma: Two case reports

**DOI:** 10.1097/MD.0000000000022478

**Published:** 2020-10-09

**Authors:** Shuang-lin Deng, Ri-hua Jin, Yi-ming Liu, Yi Jing, Yi Guan

**Affiliations:** a Department of Oncological neurosurgery, First Hospital of Jilin University; b Department of Pharmacy, Second Hospital of Jilin University, Changchun, Jilin Province, China.

**Keywords:** case report, diagnosis, epileptic seizure, inflammatory granuloma, pleomorphic xanthoastrocytoma

## Abstract

**Rationale:**

Pleomorphic xanthoastrocytoma (PXA) is a rare low-grade glial neoplasm of the central nervous system, which is difficult to distinguish from other neoplastic and non-neoplastic entities. Herein, we report 2 cases of PXA that had been misdiagnosed as an inflammatory granuloma.

**Patient concerns:**

The first case was a 22-year-old man who originally presented with a generalized seizure 7 years previously. Magnetic resonance imaging (MRI) revealed a lesion in the right parietal lobe, leading to a diagnosis of inflammatory granuloma. The second case was a 43-year-old man who presented with repeated generalized seizures. MRI revealed a nodular lesion in the left temporal lobe. The magnetic resonance spectrum showed elevated Cho and NAA peaks and a decreased Cr peak. An inflammatory granuloma was suspected.

**Diagnosis:**

After surgical treatment, histopathological examination revealed PXA.

**Interventions:**

In the first case, after 10 months of anti-inflammatory treatment, the lesion was significantly reduced in size. During the following 7 years, the patient experienced generalized seizures 3 to 4 times annually. To control intractable epilepsy, the lesion was resected. In the second case, conservative treatment provided no benefit, and then the lesion was resected.

**Outcomes:**

In the first case, during a follow-up period of 14 months, the patient was seizure-free with no tumor recurrence. In the second case, after a 6 months of follow-up, the patient remained seizure-free with no tumor recurrence.

**Lessons:**

The preoperative differential diagnosis of PXA is challenging due to the nonspecific symptoms and imaging manifestations. Considering the potential risk of malignant transformation of PXA, early surgery should be highlighted, and gross total resection is associated with a favorable prognosis

## 1 Introduction

Pleomorphic xanthoastrocytoma (PXA) is a rare low-grade glial neoplasm of the central nervous system. PXA was first described as a pathologically distinct entity by Kepes and coauthors in 1979.^[1]^ Since 1993, PXA has been included in the World Health Organization (WHO) classification of tumors of the central nervous system.^[2]^ In the 2007 edition of the WHO classification, PXA was regarded as a Grade II benign glioma, with certain cases exhibiting signs of anaplasia,^[3]^ while in the 2016 edition, PXAs with anaplastic features were upgraded to Grade III “*anaplastic PXAs*”, which have a significantly poorer prognosis compared with the classical counterparts.^[4,5]^

Due to the rarity of PXAs, the clinical and radiological characteristics have yet to be fully understood. In the limited relevant literature, PXA is generally described as a well-circumscribed mass with hypo- to iso-intensity on T1-weighted imaging and hyperintensity on T2-weighted imaging, and long-standing seizures represent the most common clinical symptoms. The preoperative diagnosis of PXA is challenging, and it is difficult to distinguish from other neoplasms, such as ganglioglioma, dysembryoplastic neuroepithelial tumor, and meningioma.^[6,7]^ Moreover, in rare cases, PXA may also be misdiagnosed as a non-neoplastic lesion, which may delay surgical treatment and even permit malignant transformation. Herein, we report 2 cases of PXA that had been misdiagnosed as an inflammatory granuloma. Additionally, the relevant literature is reviewed.

## 2 Case presentation

### 2.1 Case 1

**Figure 1:**
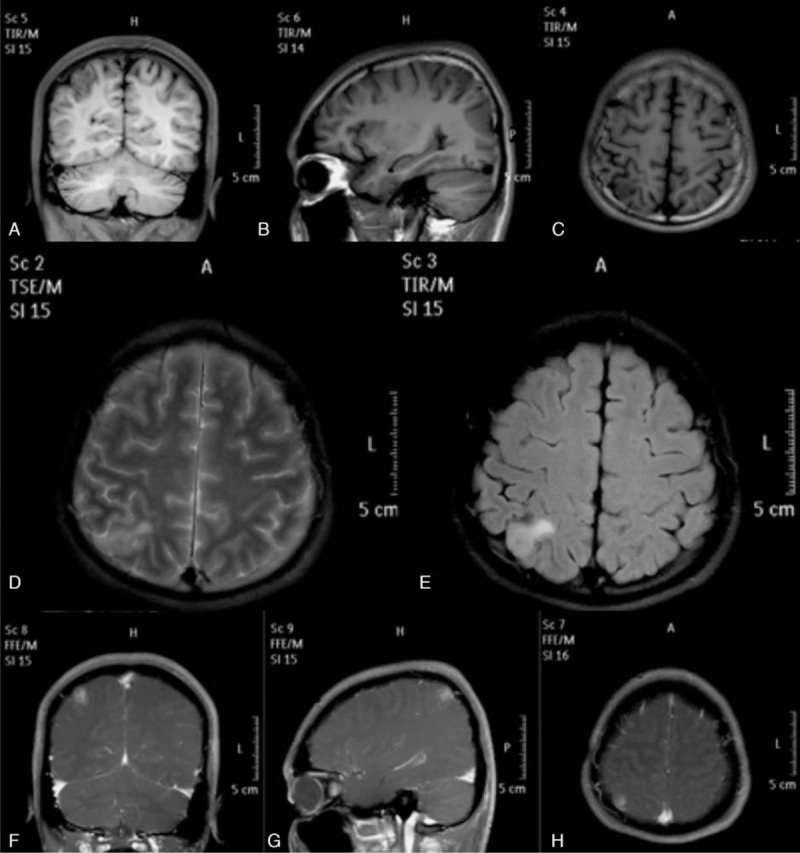
Preoperative MRI in Case 1. Coronal (A), sagittal (B), and axial (C) T1-weighted imaging showed a hypointense lesion in the right parietal lobe. The lesion was hyperintense on T2-weighted imaging (D) and FLAIR imaging (E). Coronal (F), sagittal (G), and axial (H) contrast T1-weighted imaging showed the lesion was heterogeneously enhanced.

**Figure 2:**
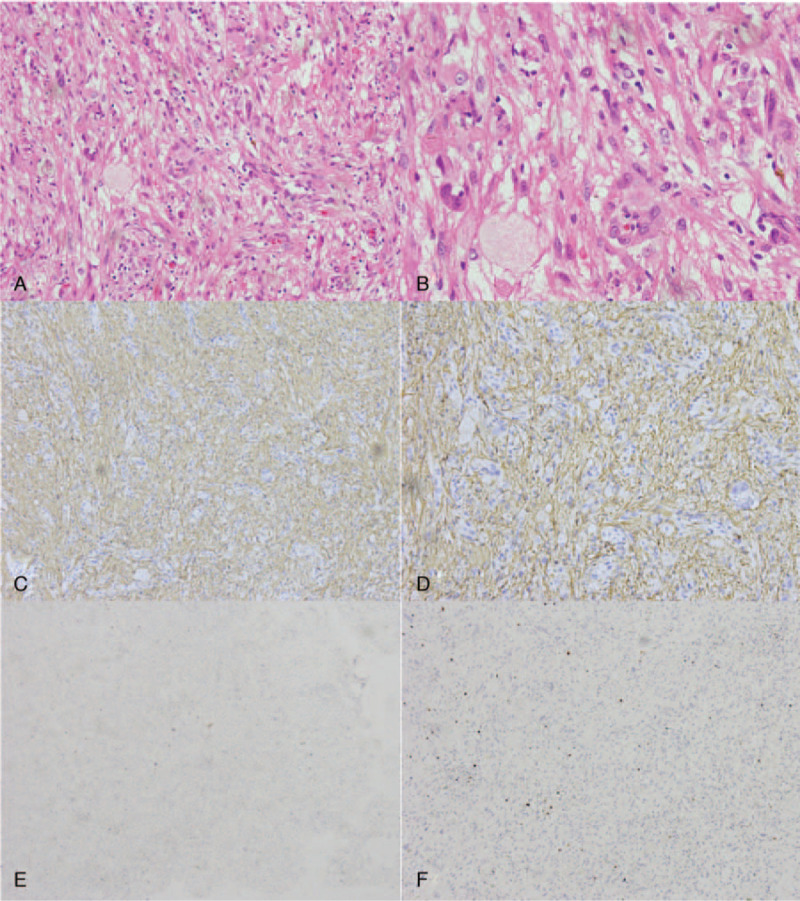
Histopathological examination in Case 1. Hematoxylin and eosin staining (A, ×200; B, ×400) showed a PXA. Immunohistochemical staining showed the tumor was positive for GFAP (C, ×200; D, ×400). The marker of proliferation Ki-67 labeling index was ∼2% (E, ×200; F, ×400).

**Figure 3:**
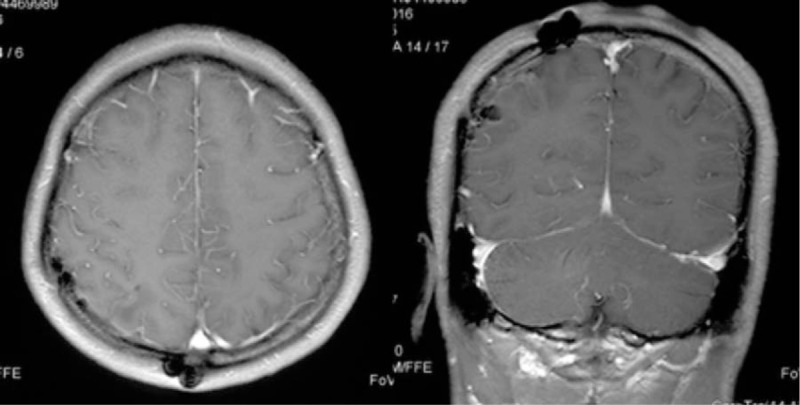
Follow-up MRI in Case 1. Fourteen months after the operation, follow-up MRI showed no tumor recurrence.

A 22-year-old man presented with a long-standing history of epileptic seizures. Approximately 7 years previously, the patient had been admitted to our department with a generalized seizure that lasted for 2 minutes. Brain magnetic resonance imaging (MRI) revealed an 8-mm-diameter, poorly-demarcated lesion in the right parietal lobe with mild peritumoral edema. The lesion showed slight hypointensity on T1-weighted imaging, hyperintensity on T2-weighted imaging, and slight hyperintensity on fluid-attenuated inversion recovery (FLAIR) imaging. After administration of Gd-diethylenetriamine penta-acetic acid (Gd-DTPA), the lesion showed heterogeneous enhancement. A diagnosis of inflammatory granuloma was suspected, and anti-inflammatory and anti-epileptic treatments were performed. Ten months later, repeated MRI demonstrated that the lesion was significantly reduced in size (diameter, 5 mm), and thus, no surgical treatment was recommended. During the subsequent 7 years, the patient experienced generalized seizures 3 to 4 times annually. On this admission, intravenous cefepime (2 g/day) and oral sodium valproate (1 g/day) were prescribed. After a 2-month pharmacotherapy, brain MRI showed the lesion remained unchanged (Fig. 1). To control intractable epilepsy, surgical resection of the lesion was performed. With the assistance of neuro-navigation and intraoperative electrophysiological monitoring, a brown soft lesion was gross totally resected. The epileptic waves dissipated 10 minutes after the resection. Histopathological examination revealed a PXA, with positive staining for glial fibrillary acidic protein (GFAP), vimentin, synapsin (Syn), S-100, p53, oligodendrocyte transcription factor 2 (Olig2), microtubule associated protein (Map2), CD68, and CD34, but negative staining for neurofilament (NF), myelin basic protein (MBP), isocitrate dehydrogenase 1 (IDH1) R132H, and Neuronal Nuclei (NeuN). The marker of proliferation Ki-67 labeling index was ∼2%, and the fraction of MGMT immunopositive tumor cells was ∼60% (Fig. 2). During a follow-up period of 14 months, the patient remained seizure-free, and no tumor recurrence was observed (Fig. 3).

### 2.2 Case 2

**Figure 4:**
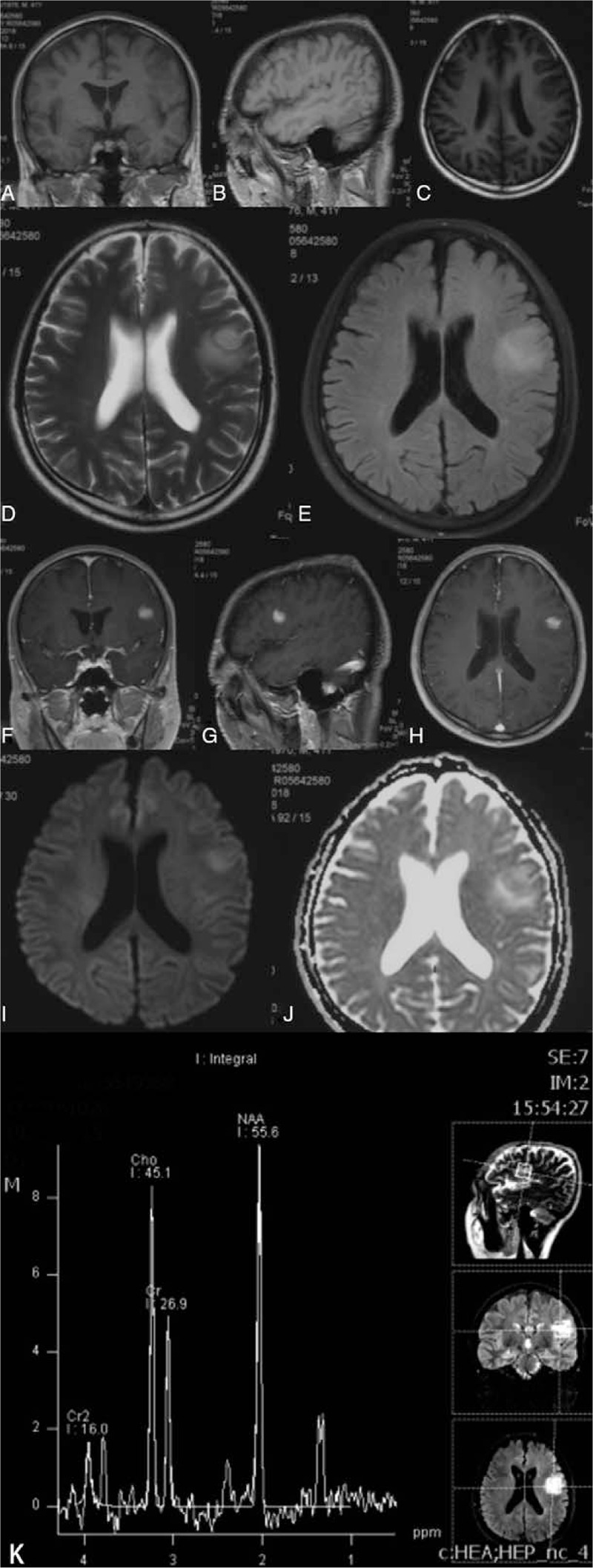
Preoperative MRI in Case 2. Coronal (A), sagittal (B), and axial (C) T1-weighted imaging showed a hypointense lesion in the left temporal lobe. The lesion showed hyperintensity with remarkable peritumoral edema on T2-weighted imaging (D) and FLAIR imaging (E). Coronal (F), sagittal (G), and axial (H) contrast T1-weighted imaging showed the lesion was markedly enhanced. The tumor showed iso- to hyperintensity with a circular hypointense core on DWI (I) and apparent diffusion coefficient imaging (J). MRS showed elevated Cho and NAA peaks and a decreased Cr peak (K).

**Figure 5:**
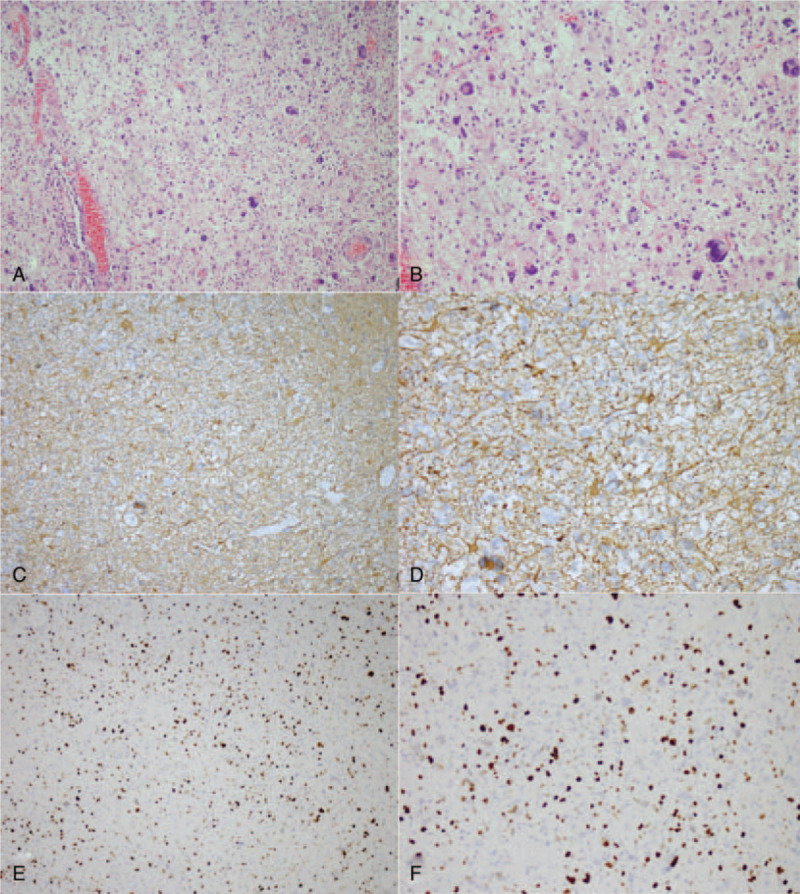
Histopathological examination in Case 2. Hematoxylin and eosin staining (A, ×200; B, ×400) showed a PXA. Immunohistochemical staining showed the tumor was positive for GFAP (C, ×200; D, ×400). The marker of proliferation Ki-67 labeling index was ∼1% (E, ×200; F, ×400).

**Figure 6:**
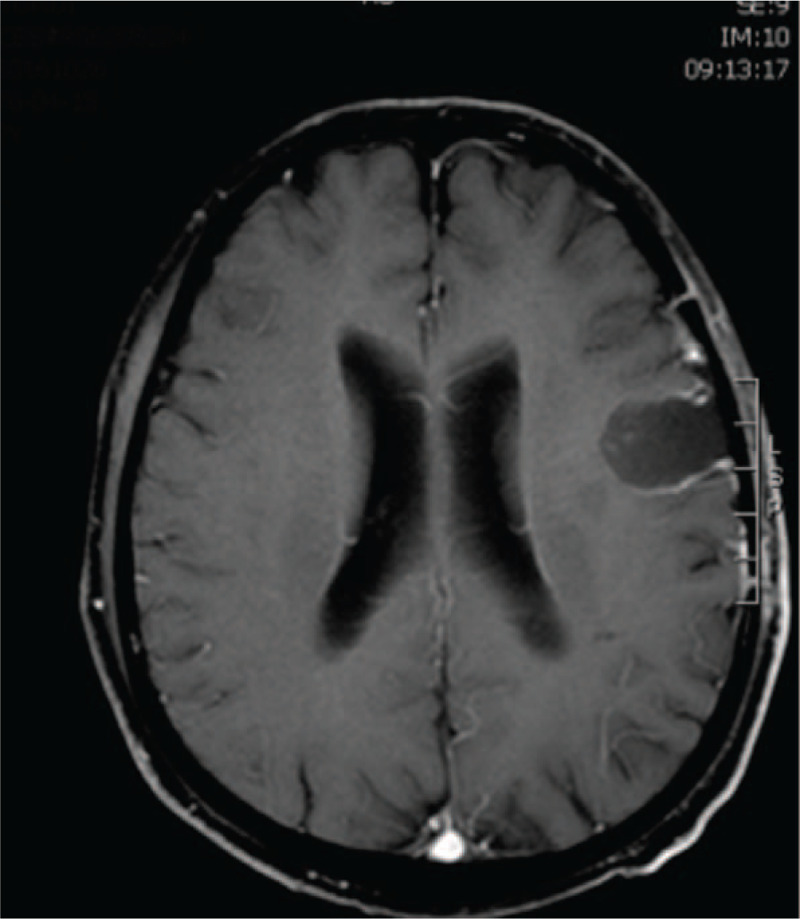
Follow-up MRI in Case 2. Six months after the operation, follow-up MRI showed no tumor recurrence.

A 43-year-old man presented to us with repeated generalized seizures occurring 5 times in the previous 10 days, with each episode lasting for about 1 minute. On admission, physical examination showed no abnormalities. Brain MRI revealed a nodular lesion in the left temporal lobe with hypointensity on T1-weighted imaging and hyperintensity on T2-weighted and FLAIR imaging; the lesion showed iso- to hyperintensity with a circular hypointense core on diffusion-weighted imaging (DWI). There was remarkable peritumoral edema. After the administration of Gd-DTPA, the lesion was markedly enhanced. The magnetic resonance spectrum (MRS) showed elevated Cho and NAA peaks and a decreased Cr peak, yielding a (Cho+Cr)/NAA ratio of 1.3 (Fig. 4). Cerebrospinal fluid examination showed an elevated immunoglobulin G (IgG) level. A suspected diagnosis of inflammatory granuloma was made, and the patient was given oxcarbazepine (600 mg/day). Five months later, brain MRI showed that the lesion remained unchanged, and surgical resection via the left frontal-temporal approach was scheduled. Intraoperatively, we found a reddish soft lesion located 1 cm beneath the cortical surface. A gross total resection of the lesion was achieved, and the patient was seizure-free postoperatively. Histopathological examination revealed a PXA, with positive staining for GFAP, S-100, and Olig2, but negative staining for MBP, EMA, and PR. The marker of proliferation Ki-67 labeling index was ∼1%, and the fraction of MGMT immunopositive tumor cells was ∼1% (Fig. 5). After a 6-month follow-up, the patient remained seizure-free, and brain MRI showed no tumor recurrence (Fig. 6).

## 3 Discussion

As PXA is a relatively rare entity, there is scarce evidence concerning the corresponding neuroimaging features. We reviewed the relevant literature and identified 14 articles that provide detailed radiological illustrations,^[7–20]^ including cystic/solid components, presence of leptomeningeal contact, hydrocephalus, peritumoral edema, calcification, signals on T1- and T2-weighted imaging, enhancement of the solid component, and enhancement of meninges. These features are summarized in Table 1. The MRI characteristics of PXAs are nonspecific, with lesions appearing hypointense on T1-weighted imaging and hyperintense on T2-weighted imaging, with contrast enhancement of the solid component. Notably, the majority of reported PXAs were located peripherally, especially in superficial areas with leptomeningeal contact. However, there is remarkable inconsistency in the presence of cystic changes and peritumoral edema among studies. Crespo-Rodríguez et al analyzed the imaging features of PXA in a large cohort of 24 cases and found that the majority of PXAs are characterized by cystic changes and marked heterogenous enhancement of the solid component without peritumoral edema.^[13]^ Although meningeal enhancement and calcification have been reported in some cases, the accurate incidence and diagnostic value of these features remain unclear. Yu et al performed another large-cohort neuroimaging study on PXAs, in which a pure solid pattern was seen in only 25% of all cases and most of the cases exhibited peritumoral edema.^[19]^ Moore et al analyzed the MRI and DWI features of PXAs in pediatric patients, and their findings also support that cystic changes and peritumoral edema are common characteristics.^[14]^ In our literature review, approximately 55% of all reported cases manifested as cystic changes, and peritumoral edema was noted in more than 60% of all cases. However, in the current 2 cases, no cystic signals were observed on MRI. Furthermore, the tumors in this case report were both peripherally located and small in size, with no remarkable space-occupying effects, supporting the diagnosis of a benign entity associated with slow growth. Preoperatively, these 2 cases were both misdiagnosed as an inflammatory granuloma. In the first case, the tumor decreased in size after anti-inflammation treatment; in the second case, MRS showed an increased NAA peak. These features misled the diagnosis and delayed surgical treatment.

**Table 1:**
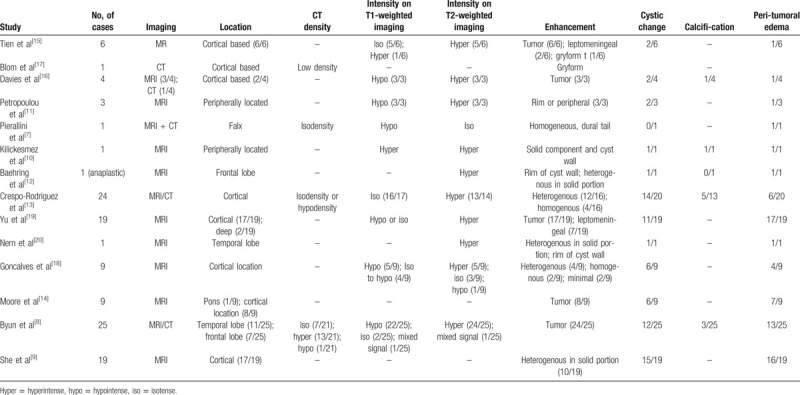
Review of previous studies on radiological features of PXA.

Studies on the radiological features of inflammatory granulomas are also limited. During the past three decades, with the advances in diagnostic modalities, the identification rate of this non-neoplastic entity has been greatly improved. Intracranial inflammatory granulomas are caused by a broad spectrum of pathogens, including bacteria, fungi, viruses, parasites, immunogens, and foreign matter.^[21,22]^ The formation of granulomas is a cell-mediated inflammatory response associated with the recruitment of lymphocytes, endothelial cells, and macrophages. Similar to intracranial tumors, inflammatory granulomas may also manifest as space-occupying effects; nevertheless, the local compression on the cerebral gyrus, parenchyma, cisterns, and ventricles is uncommon, and perilesional edema is often unremarkable. In cases without significant compression, surgical resection is not the first choice of treatment, and it should only be considered when conservative therapies fail to alleviate clinical symptoms. The definitive etiology of inflammatory granuloma is difficult to determine and depends on biopsy or postoperative histopathology.^[23]^ According to the various causes, the optional pharmacotherapeutic regimens include antibiotics, steroids, and immunomodulators. Al-Afif et al identified 40 patients with intracranial inflammatory lesions from 3066 reports of histopathological examinations and proposed that the diagnostic and therapeutic value of surgery should be highlighted.^[24]^ In our cases, the clinical courses were much more complex, and the tumors responses to antibiotics and MRS parameters highly suggested a benign non-neoplastic nature.

Surgical resection is the mainstay treatment for PXA. Epileptic seizures are common clinical manifestations in patients with PXA, and complete resection of the tumor has been reported to be an effective approach to control medically intractable epilepsy. The overall prognosis of PXA is favorable; nevertheless, monitoring for recurrence and malignant transformation is warranted. According to the existing literature, the recurrence rate of PXA following surgical resection is about 30% within 5 years and 40% within 10 years, and the overall survival rate is about 80% at 5 years and 70% at 10 years.^[1,25,26]^ Recently, Mallick et al performed a meta-analysis of PXA involving 167 previously reported cases, in which the estimated median overall survival was 209 months, and the estimated median progression-free survival was 48 months. Moreover, they found that gross total resection was associated with a significantly better survival outcome compared with subtotal resection.^[27]^ Ida et al analyzed the natural history and long-term follow-up outcomes of PXA in 74 patients. In their large-cohort study, the estimated 5-year recurrence-free survival for gross total resection (GTR) patients was 84.9%, while an evolution into a more aggressive form was observed in almost half of the recurrent PXAs.^[28]^ Additionally, some scholars noted that 15% to 20% of PXAs, which are less pleomorphic and more diffusely infiltrative, may undergo progressive anaplastic transformation.^[29]^ The mechanism underlying the anaplastic transformation of PXAs remains unclear. Recent studies found that promoter mutations in the telomerase reverse transcriptase (TERT) gene and DNA methylation alterations may be potential drivers of anaplastic transformation in PXAs.^[30,31]^ Considering the potential risks of malignant progression, the necessity of early surgical intervention should be emphasized.

## 4 Conclusions

We report the clinicoradiological and histopathological profiles of 2 cases of PXA mimicking inflammatory granuloma. PXA typically have a superficial location, involving the leptomeninges and cerebrum, most commonly in the temporal lobe. However, some cases with a long disease course and very slow progression could be confused with inflammatory lesions requiring differential diagnosis. PXAs exhibit potential risk for malignant transformation, and early surgical treatment should be highlighted. Gross total resection is associated with a favorable prognosis.

## Author contributions

Shuang-lin Deng and Ri-hua Jin designed/performed most of the data and literature analysis and wrote the manuscript

Yi-ming Liu contributed to the designing of the antibiotic treatment and participated in the differential diagnosis of the cases

Yi Jing contributed to the patient care and data collection

Yi Guan was the chief neurosurgeon responsible for the performed surgeries and the overall designing of the manuscript.

## Abbreviations

Abbreviations: DWI = diffusion-weighted imaging, FLAIR = fluid-attenuated inversion recovery, Gd-DTPA = Gd-diethylenetriamine penta-acetic acid, GFAP = glial fibrillary acidic protein, IDH1 = isocitrate dehydrogenase 1, Map2 = microtubule associated protein, MBP = myelin basic protein, MRI = magnetic resonance imaging, MRS = magnetic resonance spectrum.NeuN = neuronal nuclei, NF = neurofilament, Olig2 = oligodendrocyte transcription factor 2, PXA = pleomorphic xanthoastrocytoma, Syn = Vimentin, synapsin, WHO = World Health Organization.
